# Air Pollution: Salt Mist Is the Right Seasoning for Ozone

**DOI:** 10.1289/ehp.116-a288

**Published:** 2008-07

**Authors:** Carol Potera

Shipping ports face a newly discovered air pollution problem—the production of the ozone precursor nitryl chloride. Nitryl chloride was detected for the first time in the lowest part of the Earth’s atmosphere by a team from the National Oceanic and Atmospheric Administration (NOAA) that was monitoring air quality in Galveston Bay to understand why nearby Houston, Texas, has one of the worst air pollution problems in the nation. Salts in ocean mists were thought to be relatively inert until the connection to ozone was uncovered. “People never before thought that nitryl chloride was important,” says James Roberts, a NOAA research chemist and the team’s coordinator.

In the summer of 2006, the researchers used chemical ionization mass spectrometry to detect trace levels of airborne chemicals, including nitryl chloride. They found that when ship exhaust plumes rich in nitrogen oxides (NO_x_) meet ocean air at night, unexpectedly high levels of nitryl chloride form due to the NO_x_ species dinitrogen pentoxide combining with chloride in sea mist. After the sun comes up, this buildup of photoactive nitryl chloride splits into chlorine atoms and nitrogen dioxide. These compounds then accelerate the production of ozone, a key component of smog.

The amount of nitryl chloride measured by Roberts and colleagues—as high as 650 ppt by volume, or about 15% of total reactive nitrogen species from ship exhaust—is much greater than that estimated by standard air pollution models, which have taken into account neither the heightened presence of nitryl chloride around ports nor its importance in forming ozone. “This preliminary study indicates that nitryl chloride chemistry could make a significant contribution—up to ten to thirty percent—to ozone production during the morning hours in Houston,” says Roberts. These results were published in the May 2008 issue of *Nature Geoscience*.

The study findings point to the need to control NO_x_ emissions from fossil fuel combustion in coastal cities, says Roberts. He adds that one solution may be to require docked ships to use local electrical power, rather than burning diesel fuel, to generate electricity. Some ports have begun to pursue this course of action [see “Ports in a Storm,” *EHP* 114:A222–A231 (2006)]. The 10-year $750-million Middle Harbor Redevelopment Project proposed by the Port of Long Beach (California) would, among other pollution mitigation strategies, provide shoreside electricity for docked vessels.

About half the world’s population lives near coastlines where industrial pollution meets ocean air, Roberts says, so nitryl chloride could play a major role in air quality worldwide. The same reaction likely occurs inland, where chloride-containing aerosols drive the chemical reaction. Inland sources of chloride include natural soil salts such as calcium chloride and de-icing compounds spread on winter roads.

“We don’t know how widespread nitryl chloride is as a source of ozone pollution,” says Roberts. The health consequences of nitryl chloride itself are unknown. As for ozone, an April 2008 report by the National Research Council, *Estimating Mortality Risk Reduction and Economic Benefits from Controlling Ozone Air Pollution*, links even short-term exposure with premature death.

“This is the first field measurement of nitryl chloride, and it’s very exciting,” says Barbara Finlayson-Pitts, a professor of chemistry at the University of California, Irvine. Experiments in her laboratory 20 years ago first showed that mixing sodium chloride with dinitrogen pentoxide in the dark generated nitryl chloride. “The data will be extremely useful for further development and application of air quality models for coastal urban areas,” she says.

## Figures and Tables

**Figure:**
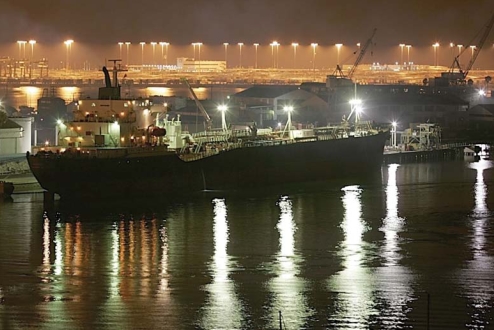
The NO_x_ in ship exhaust combines with chloride in sea mist to produce a potent ozone precursor

